# Did the COVID-19 pandemic help or hurt Donald Trump’s political fortunes?

**DOI:** 10.1371/journal.pone.0247664

**Published:** 2021-02-24

**Authors:** Joshua Hart

**Affiliations:** Department of Psychology, Union College, Schenectady, New York, United States of America; Sogang University (South Korea), REPUBLIC OF KOREA

## Abstract

The COVID-19 pandemic likely had an effect on the outcome of the 2020 US presidential election. Was it responsible for the defeat of incumbent President Donald Trump? The present study makes an initial attempt at, and provides a model for, understanding the pandemic’s influence on Trump support. The study employed a mixed experimental and correlational design and surveyed separate samples of adults (N = 1,763) in six waves beginning March 23, 2020 and ending June 1, 2020. Participants were randomly assigned to report their Trump support either before or after being reminded of the pandemic with a series of questions gauging their level of concern about it. Results revealed complex and dynamic effects that changed over time. Depending on survey wave, the pandemic seems to have lowered Trump support among Democrats, while (marginally) raising it among independents. Republicans’ reactions also changed over time; of particular note, Republicans who were more concerned about the pandemic reported higher Trump support after being reminded of the pandemic in its early stages, but this effect reversed by the time the economy began reopening (coinciding with a dip in Trump’s approval ratings). Although the correlational results in the present study did not converge neatly with the experimental results, the combined experimental and correlational approach has the potential to increase researchers’ confidence in the causal effects of salient national and international events on political attitudes.

## Introduction

The 2019–2021 coronavirus pandemic has widespread psychological, sociological, and political implications. Among these is the likely effect on people’s support for incumbent leaders, including United States President Donald Trump. Social science theories can be used to generate multiple (conflicting) predictions about this, and although the 2020 U.S. presidential election results are known, it is not clear what, if any, role the pandemic played in Trump’s defeat, and there is evidence to support both a negative effect of the pandemic on Trump support [1], and a positive one [2] prior to the election. The present study provides additional data that social scientists might use to help understand, retrospectively, the COVID-19 pandemic’s effect on Trump support among the US electorate, and prospectively, the potential effects of future disasters on major elections. More generally, the study provides a relatively novel methodological approach that can help researchers make causal inferences about the influence of large-scale naturalistic events on political outcomes.

### The politics of disaster: Theories and research

From a psychological perspective, a global pandemic involving a novel, highly contagious, and lethal virus, could be construed in several ways. Such a crisis, with its startling death toll, would likely represent a massive ongoing mortality salience induction, creating widespread death anxiety. According to terror management theory (e.g., [3]), this would tend to increase defensive political polarization. Existential fears lead people to seek refuge in their beliefs and attitudes about the world, which provide a comforting sense of meaning and understanding, order, structure, predictability, and control [3,4]. In other words, existential concerns lead people to cling more tenaciously to assuaging worldviews. In this case, one would expect the pandemic to increase conservatives’ Trump support, while decreasing liberals’ Trump support.

Alternatively, the uncertainty aroused by the pandemic might result in a generalized “conservative shift” by increasing people’s need to support the political status quo, as some motivated social cognition models would predict [e.g., 5] (cf. the “rally ‘round the flag effect” [6]). For example, some evidence suggests that the September 11, 2001 terrorist attacks on the U.S. and the ensuing precarious national security climate could have bolstered support for incumbent George W. Bush’s reelection [7,8]. The conservative shift phenomenon is also consistent with some theorizing about the psychological roots of political conservatism; for example, conservatives’ emphasis on robust domestic law enforcement and national security matches well with beliefs that the world is a fundamentally dangerous and unruly place [9]. According to this logic, the unsettling long-term disruptive effect of a pandemic might be expected to cause a bump in Trump support across the political spectrum, as people gravitate toward the perceived comfort of a continuation of stable governance, especially when led by a president whose party (and personality) are associated with strength, law, and order.

These conflicting schools of thought suggest the possibility that the effects of the pandemic on Trump support, rather than being a simple and predictable response to psychological unease, might be more nuanced and dependent on contextual factors, as political science work suggests. For example, Gasper and Reeves [10] distinguished between a “responsive electorate” phenomenon, in which constituents reflexively punish leaders for negative events beyond their control, and an “attentive electorate” phenomenon, in which constituents effectively discern the quality of leaders’ responses to such events and calibrate their sentiment accordingly. Gaspar and Reeves found evidence for both processes, but concluded that there is stronger evidence for an attentive electorate.

One recent historical example may be particularly instructive as to the intricacies of assessing the effect of disasters on support for leaders. Immediately prior to the 2012 U.S. presidential election, the massive tropical cyclone “Sandy” made landfall, causing much speculation as to its effect on the election results. Some data suggest that in the early aftermath of the storm—when media reports showed Obama exhibiting leadership of the disaster recovery efforts, including a highly publicized collaboration with New Jersey’s Republican Governor Chris Christie—Obama received increased support, especially among conservatives [11]. This could reflect the rallying of some parts of the electorate around the incumbent during a time of uncertainty, and it is also consistent with an attentive electorate explanation. However, by election day, when news reports increasingly featured the devastating consequences of the storm, voters for whom it was salient expressed lowered support for Obama [11], suggesting that, in a sense, he was being blamed for the fallout, consistent with a conservative shift phenomenon or “blind retrospective” voting.

However, an additional factor merits consideration, particularly in the polarized political climate that characterized 2012 (and 2020): partisanship. Consistent with a terror management or “worldview defense” analysis, Heersink et al. [12] argue that voters’ tendency to rally around versus blame leaders in the wake of a disaster depends on voters’ preexisting political leanings. Indeed, Heersink et al. found evidence for this both in historical data and in their analysis of responses to support for Obama after Sandy. Specifically, Sandy appeared to have hurt Obama’s standing among voters in counties that were initially disposed against him, but not in Obama-friendly counties. (Heersink et al. refer to this phenomenon as “partisan retrospection”).

Partisan retrospection in the context of disasters and crises is also consistent with what we know about how partisanship influences information processing in a more general sense. Namely, through a combination of confirmation bias, laziness, inattention, and motivated reasoning, partisans tend to disproportionately notice, encode, and remember information that is consistent with or supportive of their political beliefs [13–15]. This phenomenon has already been found to apply to information regarding COVID-19, which became a politically polarized issue relatively early in the pandemic, influenced in part by messaging by political leaders [16]. Therefore, in the present context, there is ample reason to expect that partisanship plays an important role.

### The “Trump virus”

The Sandy-Obama example is illuminating in at least two respects, not least because it is relatively recent. First, it suggests that voter responses to a leader during a time of disaster could be highly variable, changing over time, as circumstances change for better or worse, as the leader responds in ways favorable or unfavorable to unfolding events, and as voters become more informed or attentive. Second, as psychological work suggests and Heersink et al. demonstrate [12], it is vital to account for the influence of voter partisanship.

The present study makes attempts to understand the COVID-19 pandemic’s effect on Trump support, 5–6 months in advance of his own re-election bid, during the (first) peak of the crisis and shortly thereafter. To account for potential changes over time, the study included 6 separate, brief surveys of ~300 US residents each. The first 5 surveys were spaced one week apart starting just days after the commencement of widespread "lockdowns" in the US. The final survey occurred 6 weeks after the end of the first series, when the nation had begun reopening the economy. Because it is impossible to conduct a true experiment (i.e., one cannot randomly assign people to a universe without the pandemic), my analytic approach follows the mixed experimental and correlational design suggested by Hart [11]. Participants were asked about their concern and exposure to media about the pandemic, along with their support for President Trump. Correlating concern and exposure to Trump support provides one way of assessing a possible effect. However, the order of the topics was counterbalanced, so that approximately half the participants were effectively primed with pandemic awareness prior to reporting their Trump support. (The correlational results in this study suggest that answering pandemic questions first did not affect the correlation between pandemic concern and Trump support, and there was no main effect of priming condition on pandemic concern.) This provides a more rigorous test of the causal effect. If the experimental and correlational findings converge, it increases confidence that there is an actual causal effect.

Because theoretical models and previous research on the effects of disasters on support for leaders are in conflict, and there was no way to predict how Trump would handle the pandemic, I did not have specific predictions about how sentiment towards him would be influenced, except that it would likely depend in some form on respondents’ political orientation.

## Method

### Participants

This research was declared exempt from review by the Human Subjects Review Committee at Union College, Schenectady, NY, USA. Informed consent was obtained by a clickable web button and data were anonymous. Responses were analyzed from a total of 1,763 participants (711 women, 1048 men, 4 “neither”) in the US who participated in a “brief opinion survey” via MTurk.com and were paid $.50 for an approximately 5-minute survey. The sample size of approximately 300 (different sets of) people per wave of the survey was set to achieve 80% power in a 3 (party affiliation: Democrat, Republican, or neither) x 2 (priming condition: Pandemic priming or not) design and an estimated “small” effect size of approximately .20. (The size of some waves was slightly under 300 due to a handful of participants failing to submit their completed surveys.) The survey was set to prevent participants from completing the survey more than once, and an analysis of their MTurk IDs suggests that this was successful for all but 2 participants, whose data from the second survey completion were deleted prior to analysis.

### Materials and procedure

The surveys were distributed on Monday afternoons (March 23, March 30, April 6, April 13, April 20 and June 1) beginning around 1:00 pm EST; each completed in less than 8 hours. Participants were randomly assigned to either the pandemic priming or non-priming condition. Those assigned to the pandemic priming condition answered questions about the pandemic before questions about Trump; those in the non-priming condition answered the two sets of questions in the opposite order.

Four pandemic questions assessed “pandemic concern”: “How much have you been following news or other media coverage of the coronavirus pandemic?”; “How much have you, or people close to you, been affected by the coronavirus pandemic?”; “How much time have you spent thinking about the coronavirus pandemic?”; and “How concerned are you about the coronavirus pandemic?” (all answered on a scale of 1 = *not at all* to 5 = *an extreme amount*). The items formed a reliable scale, α = .77 (*M* = 3.45, *SD* = .74).

Three questions tapped support for President Trump: “I think that President Trump is doing a good job on the economy”; “I think President Trump is an overall good president”; and “I will vote to re-elect President Trump in the November 2020 election.” Participants indicated their agreement on a 1 (*disagree strongly*) to 7 (*agree strongly*) scale. (I did not ask questions about handling of the pandemic to avoid priming it in the non-priming condition.) The aggregate of these items was highly reliable, α = .96 (*M* = 3.26, *SD* = 2.13).

Finally, participants gave their age, gender, political orientation (1 = *extremely liberal*; 9 = *extremely conservative*; *M* = 4.05, *SD* = 2.19) and political party affiliation (*Democrat*, *Republican*, or *neither;* hereafter referred to as “independent”).

## Results

### Experimental analysis

To assess whether the relative salience of the pandemic would influence Trump support at each wave of the study, I first conducted a 3 (party affiliation: Democrat, Republican, or independent) x 2 (priming condition: Pandemic priming or not) x 6 (survey wave: 1–6) Analyses of Variance (ANOVAs). (All analyses included gender and age as covariates, as these are widely known predictors of Trump support).

There was a strong main effect of political affiliation, such that Republicans expressed higher Trump support than Democrats and independents, *F*(2, 175) = 1212.37, *p* < .001, η_p_^2^ = .41. On the 7-point scales, Republicans (n = 467) registered a mean approval rating of 5.45 (*SD* = 1.39), compared to Democrats’ *M* = 2.18 (*SD* = 1.61) and independents’ *M* = 3.07 (*SD* = 1.90). In line with the expectation that pandemic priming might have different effects depending on political affiliation and time, there was also a 3-way interaction, *F*(10, 1721) = 2.28, *p* = .01, η_p_^2^ = .013. To probe the interaction, I conducted separate 2 (pandemic priming) x 6 (survey wave) ANOVAs within each category of political affiliation (see Table 1 for descriptive statistics).

**Table 1:** Trump support as a function of date and political affiliation.

Date	Democrat *M*(*SD*)	Republican *M*(*SD*)	Independent *M*(*SD*)
March 23	1.93(1.43)	5.71(1.41)	2.79(1.67)
March 30	2.06(1.51)	5.35(1.44)	3.42(1.99)
April 6	2.24(1.59)	5.77(1.06)	3.06(2.00)
April 13	2.31(1.73)	5.41(1.35)	3.09(1.93)
April 20	2.27(1.68)	5.53(1.42)	2.94(1.86)
June 1	2.26(1.74)	5.22(1.51)	3.10(1.95)

#### Democrats

Among Democrats, pandemic priming interacted with survey wave, *F*(5, 850) = 2.23, *p* = .05, η_p_^2^ = .013. Separate ANOVAs conducted for each wave revealed that on March 23, pandemic priming decreased Trump support *F*(1, 167) = 3.88, *p* = .05, η_p_^2^ = .02 (*M* = 2.18, *SD* = 1.63 vs. *M* = 1.74 *SD* = 1.23); whereas just a week later (March 30) there was no effect. There was also no effect on April 6, but there was a noticeable trend consistent with the negative effect of pandemic priming on March 23, *F*(1, 153) = 2.30, *p* = .13, η_p_^2^ = .015 (*M* = 2.45, *SD* = 2.48 vs. *M* = 2.06, *SD* = 2.04). There were also no effects on April 13 or April 20. However, by the June 1 “reopening” survey, the negative trend had returned, *F*(1, 127) = 3.33, *p* = .07, η_p_^2^ = .03 (*M* = 2.54, *SD* = 1.80 vs. *M* = 1.97, *SD* = 1.64).

In all, these results are consistent with a modest negative impact of the pandemic on Democrats’ Trump support; however, the effect waxed and waned across the first 5 weeks of the pandemic and subsequently.

#### Republicans

Among Republicans, there was no effect of pandemic priming, but there was an effect of survey wave, *F*(5, 453) = 2.31, *p* = .04, η_p_^2^ = .025. Simple effects comparisons showed that Republicans’ Trump support was significantly lower on June 1 (*M* = 5.14) compared with on March 23 (*M* = 5.75, *p* = .01).

#### Independents

As among Democrats, among independents, pandemic priming interacted with survey wave, *F*(5, 414) = 2.52, *p* = .03, η_p_^2^ = .03. Separate ANOVAs for each wave revealed that on March 23, there was no effect of pandemic priming, but a week later on March 30, it decreased Trump support *F*(1, 73) = 4.86, *p* = .03, η_p_^2^ = .06 (*M* = 3.92, *SD* = 1.89 vs. *M* = 2.97, *SD* = 1.97).

There was again no effect on April 6, but on April 13, pandemic priming actually *increased* Trump support *F*(1, 64) = 4.00, *p* = .05, η_p_^2^ = .059 (*M* = 2.61, *SD =* 1.73 vs. *M* = 3.58, *SD* = 2.03). There was again no effect on April 20. There was no significant effect in the June 1 “reopening” survey, but there was a noticeable trend consistent with the positive effect of pandemic priming observed on April 13, *F*(1, 66) = 1.78, *p* = .19, η_p_^2^ = .026 (*M* = 2.86, *SD* = 1.89 vs. *M* = 3.50, *SD* = 2.02).

In all, these results suggest that among independents, the first couple months of the pandemic had mixed implications for Trump support. However, excluding the negative effect observed on March 30, overall, pandemic priming seems to have marginally increased independents Trump support, *F*(1, 347) = 3.31, *p* = .07, η_p_^2^ = .009 (*M* = 2.81, *SD* = 1.80 vs. *M* = 3.18, *SD* = 1.94).

### Correlational analysis

To determine whether the results of experimental pandemic priming would converge with correlational results, I conducted regression analyses predicting Trump support as a function of concern about the pandemic. I performed these regressions separately within each political affiliation category, and, for Democrats and independents, I only included the days for which there was an effect of pandemic priming or a sizeable trend in the same direction. For Republicans, who showed no effect of pandemic priming, I included all days to determine if there was any hint of an effect in the correlational data, but I analyzed the data from waves 1–5 separately from wave 6, because Republicans’ Trump support was significantly lower in the final survey. To account for possible effects of pandemic priming, in all analyses I included a dummy variable (priming = 1; control = 0) and an interaction term (priming x concern).

#### Democrats

Confirming the experimental results, pandemic priming reduced Trump support (*β* = -.16, *p* = .001), but there was no significant association between coronavirus concern and Trump support (*β* = .07, *p* = .15), and no interaction (*β* = -.002, *p* = .97).

#### Republicans

**Fig 1:**
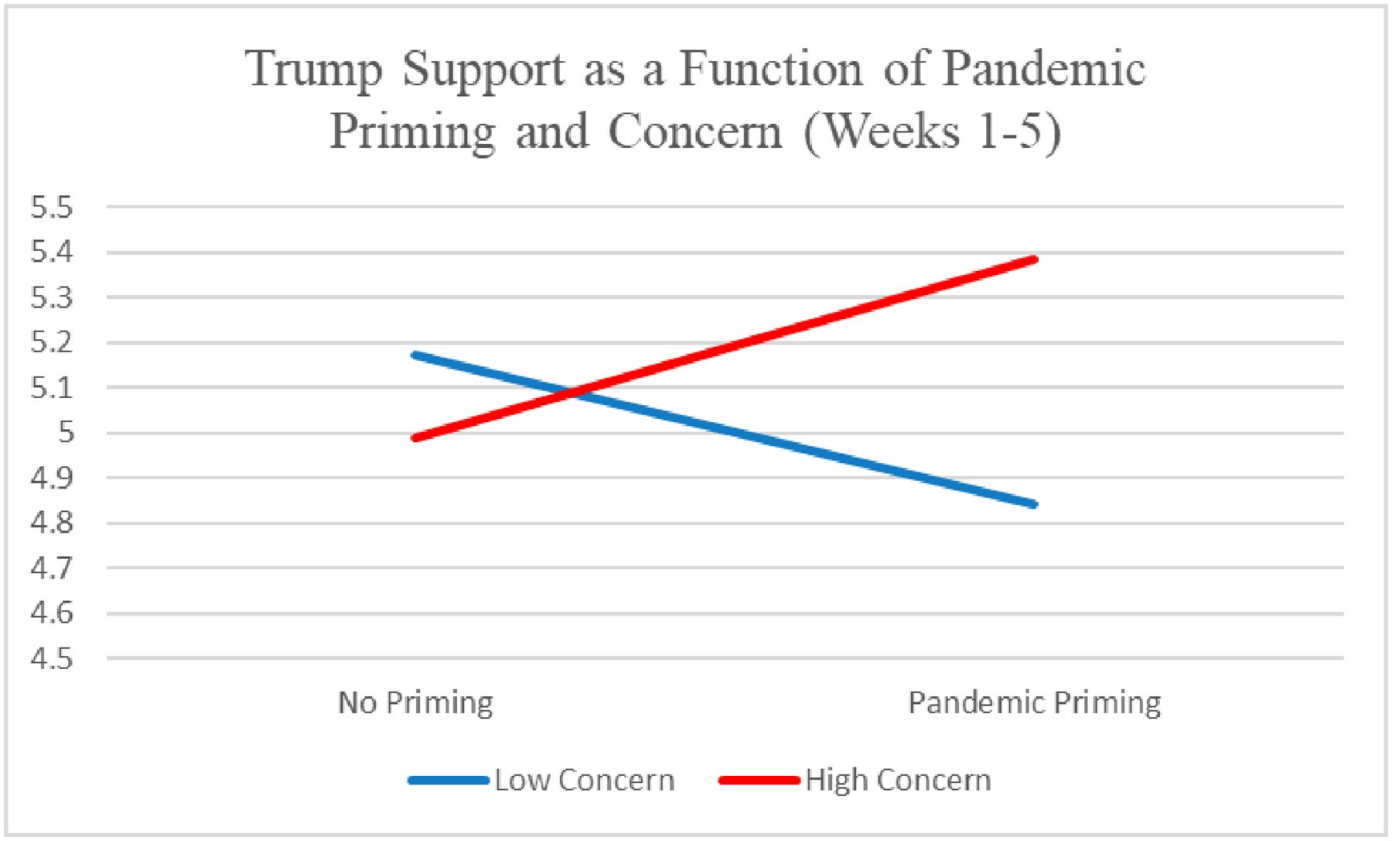
Republican Trump support as a function of pandemic priming and concern (weeks 1–5).

There was an interaction between pandemic priming and concern, *β* = .19, *p* = .01. As seen in Fig 1, the interaction suggests that Republicans highly concerned about the pandemic became more supportive of Trump after being reminded of the pandemic, whereas Republicans with lower concern became less supportive.

**Fig 2:**
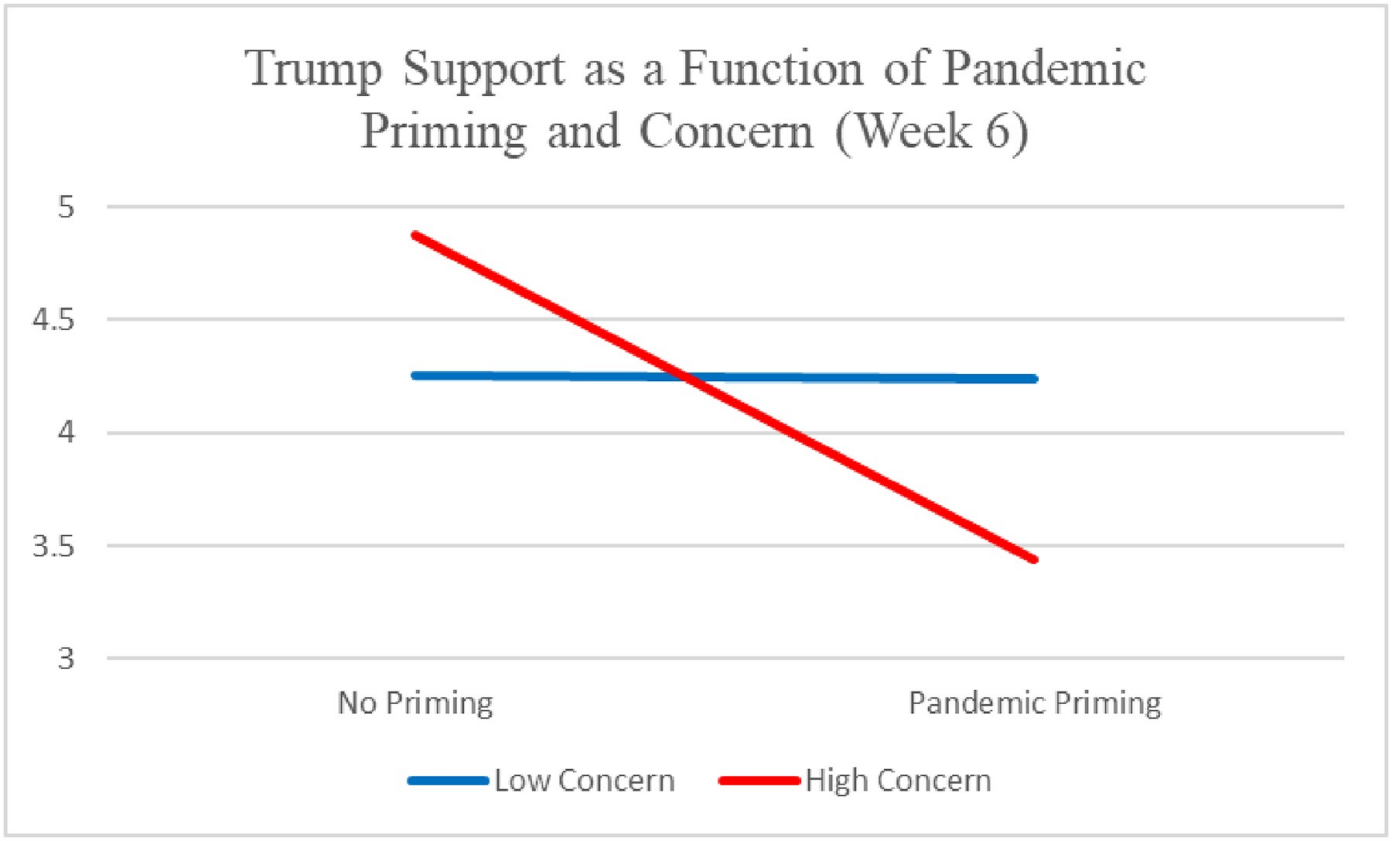
Republican Trump support as a function of pandemic priming and concern (week 6).

In the June 1 data, two interesting findings emerged. First, when controlling for pandemic concern (which the ANOVAs did not) revealed that pandemic priming reduced Trump support, *β* = -.24, *p* = .03. However, this was qualified by an interaction with pandemic concern, *β* = .27, *p* = .04. As seen in Fig 2, the negative effect of pandemic priming only occurred among Republicans higher in pandemic concern.

Despite no correlation between pandemic concern and Trump support, these findings add insight by revealing a previously undetected negative effect of pandemic priming among Republicans surveyed on June 1, which helps account for the lower overall level of Trump support found on that day.

#### Independents

Because there were mixed effects of pandemic priming among independents across waves of the study, I conducted separate analyses for April 13 and June 1 (days when pandemic priming significantly or marginally increased Trump support) and March 30. Confirming the experimental results, pandemic priming increased Trump support on the former days *β* = .19, *p* = .02. Surprisingly, in light of this main effect, pandemic concern predicted lower Trump support, *β* = -.24, *p* = .004. There was no interaction, and no correlation on March 30.

## Discussion

These results underscore that the effect of large-scale, anxiety-producing events on attitudes toward leaders is likely to be complex and dynamic. (This might be particularly true when the leaders in question tend to be polarizing.) It is not the case that a national emergency will necessarily lead to scapegoating of leaders, or to rallying support behind them; nor are the effects likely to be uniform across the political spectrum or across time.

In the present study, among Democrats for whom the COVID-19 pandemic was made salient, it seems clear that the effect on Trump support was either neutral or negative. This is consistent with either a scapegoating (i.e., blind retrospection) or worldview-defense (i.e., partisan retrospection) phenomenon and is not consistent with a conservative shift or a “rally ‘round the flag” effect.

Among Republicans, it seems that the effect depended on the extent of individual concerns about the pandemic and attentiveness to media coverage of it. But even that effect was not stable over the 9-week duration of this study. In the early stages of the pandemic lockdowns, concerned and attentive Republicans for whom it was salient reported elevated Trump support, but by the June 1 reopening, that tendency had reversed, perhaps due to disapproval with Trump’s handling of the pandemic over time (and potentially compounded by another salient national event, the police killing of Minnesota resident George Floyd and subsequent widespread protests). On June 1, approval of Trump’s handling of the pandemic response was considerably lower than during the first five weeks of the study in late March and early-mid April (e.g., according to Fivethirtyeight.com, the net approval rating was -10.5 on June 1, compared with an average of +.82 on the other 5 days of the study), when he was suggesting that the country might return to normal by the Easter holiday. Overall, it appears that the pandemic initially caused a “rally ‘round the flag” or worldview defense/partisan retrospection effect (or both) among Republicans, but that widespread criticism of Trump’s performance might have reversed these effects by June 1, consistent with an attentive electorate model.

Among independents, for whom the pandemic was salient, there was a tendency toward increased Trump support, after an early reaction in the opposite direction. Oddly enough, though, pandemic concern among independents was actually inversely related to Trump support. It is difficult to reconcile these findings. Perhaps for independents—who are likely conflicted in their Trump attitudes—that the nonconscious or indirect effects of the pandemic are different than the effects of pandemic salience. It may be that when they are actively thinking about the pandemic, they “rally ‘round the flag,” or shift to more conservative views. It is not clear why those tendencies would be opposite when simply correlating pandemic concern with Trump support. It could be that there is a third variable at play—for example, concerned individuals might represent a different “type” independent, or perhaps they are more generally anxious. Or, the correlation could be spurious. Future research should test these possibilities by measuring individual differences beyond demographic variables.

Fortunately, despite some complexity interpreting the present results, they bear clear implications for future research. When applying an experimental-priming approach (or a correlational one, for that matter) to examining the effect of national emergencies or other widely publicized and experienced situations, it is important to take into account not only individuals’ political leaning, but also to collect multiple samples over time instead of a one-shot survey. Moreover, opinion surveys focused on “pure” assessment of Trump support levels should avoid incidentally priming respondents with sensitive topics by asking questions about them prior to questions about Trump support.

### Strengths, limitations, and future directions

This study has some strengths worth emphasizing. First, it is methodologically innovative by combining a traditional tracking-survey approach with an experimental priming approach. This allows for multiple analytic strategies that can potentially provide converging results. Second, it probably generated conservative estimates of the observed effects. The COVID-19 pandemic has likely been relatively salient for most Americans since it began, and was probably near a ceiling during the period of this study; a subtle experimental prime would not be expected to have large additional effects—particularly when the dependent variable, Trump support, is known to be extremely stable.

Future research should address some of the limitations of the present study. First, there are some limitations on generalizability, as with any single sampling approach. Because the effects found here were modest in size, and MTurk samples tend to underrepresent conservatives (although this may not be true for the period covered in this study; see [17]), older adults, and are less diverse, larger sample sizes would be necessary to test for interaction effects involving individual-difference variables. However, despite its known limitations, there is empirical support the use of MTurk for research on political ideology [18], and for the conclusion that results from MTurk research across a wide variety of research topic areas usually survives validity checks [19–22].

Second, future research should include manipulations or measures that will allow for more direct testing of mechanisms (e.g., worldview defense) and differentiation of them (e.g., distinguishing between a narrow partisan phenomenon versus a broader “rally ‘round the flag” one versus a conservative shift). Third, research should take into consideration publicly available media and polling data that might shed light on the situational reasons for shifting reactions over time.

The present study provides data that can be used alongside other sources to draw inferences about the likely impact the pandemic had on the November 2020 U.S. presidential election. Future studies in the mold of the present one, ideally with a more comprehensive theory-testing focus, could be quite fruitful in contributing to understanding the effects of major national and world events on political attitudes and consequent voting behavior.

## Supporting information

S1 File (SAV)
